# Alkylated Polycyclic Aromatic Hydrocarbons Are the Largest Contributor to Polycyclic Aromatic Compound Concentrations in the Topsoil of Huaibei Coalfield, China

**DOI:** 10.3390/ijerph191912733

**Published:** 2022-10-05

**Authors:** Yahui Qian, Zhenpeng Xu, Xiuping Hong, Zhonggeng Luo, Xiulong Gao, Cai Tie, Handong Liang

**Affiliations:** 1 State Key Laboratory of Coal Resources and Safe Mining, Beijing 100083, China; 2 College of Geoscience and Surveying Engineering, China University of Mining and Technology, Beijing 100083, China; 3 College of Life Sciences, Huaibei Normal University, Huaibei 235000, China; 4 College of Chemical and Environmental Engineering, China University of Mining and Technology, Beijing 100083, China

**Keywords:** PACs, APAHs, topsoil, petrogenic source, carcinogenic risk, Huaibei coalfield

## Abstract

Alkyl polycyclic aromatic hydrocarbons (APAHs) are more toxic and persistent than their parent compounds. Here, the concentrations, composition profiles, and spatial distribution of polycyclic aromatic compounds (PACs) in 127 topsoil samples from Huaibei coalfield were analyzed. The PAC concentrations in different functional areas were significantly different: mining area > industrial area > residential area > agricultural area. APAHs were the major contributors to PACs, accounting for 71–83% of total PACs. Alkylnaphthalenes and alkylphenanthrenes were the primary APAH components, accounting for 83–87% of APAHs. Principal component analysis showed that petrogenic source, coal and biomass combustion, and vehicle emissions were the primary sources of PACs. By comparing the fingerprint information of soil, coal, and coal gangue, it was hypothesized that the petrogenic source of PAC pollution in typical mining areas and surrounding areas are coal particle scattering and coal gangue weathering. Some coal mining and industrial areas potentially pose risks to children, whereas others do not. There are limited evaluation criteria for alkyl PAHs; hence, the estimated risk is likely lower than the actual risk. In addition to the conventional 16 PAHs, it is critical to consider a broader range of PACs, especially APAHs.

## 1. Introduction

Polycyclic aromatic compounds (PACs) are a group of organic pollutants with high biological toxicity and persistency. The group contains unsubstituted polycyclic aromatic hydrocarbons (PAHs) and their derivatives, such as alkylated PAHs, oxygenated PAHs, nitrated PAHs, and hydroxy-PAHs [1,2]. Among them, alkyl polycyclic aromatic hydrocarbons (APAHs) have higher environmental concentration, stronger toxicity, and a more significant environmental accumulation effect than parent PAHs [3,4,5,6]. In general, APAHs constitute a high proportion of total PACs [4,7,8,9]. Additionally, APAHs are commonly detected in food from energy extraction areas and are transmitted through the food chain [3]. Among the hundreds of PAHs, 16 PAHs frequently found in environmental samples were designated as ‘priority pollutants’ by the United States Environmental Protection Agency (USEPA) [10,11,12,13,14,15], and they have been the focus of previous studies. However, this limited scope may underestimate the actual toxicity risk. Therefore, the assessment of APAHs in the environment deserves more attention. This study defines PACs as consisting of 16 PAHs and APAHs. 

Coal resource utilization and environmental protection is a critical global issue. Considerable research has focused on toxic metal/metalloid release from coal [16,17], but few reports focus on PAC pollution in the soil of coal mining areas. Coal and coal gangue are rich in PACs [18,19,20]. Coal mining activities, mining waste, and coal-processing residues are significant sources of PACs in coal mining areas [21,22]. The identification methods of PAC sources mainly include diagnostic ratios, principal component analysis (PCA), and positive matrix factorization (PMF) [10]. Principal component analysis is often used to analyze the origin of PACs in soil. It mainly uses the orthogonal transformation method to extract the principal components under different factor loads. Principal components are explained by the loadings of PACs and are used to determine the type of source emissions. Soil is one of the main reservoirs of PAHs [23,24]. Coal-related soil pollution can have a long residence time due to significant pollutant accumulation and environmental release periods. Coal-polluted soil overlaps with areas of intensive human activities on a spatial scale, therefore posing potential risks to human health, e.g., cancer risk. However, previous cancer risk assessments for PACs in soil were usually limited to 16 PAHs and lacked APAHs.

In this study, gas chromatography–mass spectrometry (GC–MS) was used to analyze the concentration, composition, source, and cancer risk of PACs in the topsoil of typical mining areas and surrounding areas in the Huaibei Coalfield, China. It was revealed that coal and coal gangue are the petrogenic sources in a typical coal mining area. This study expands the analysis of PAC species in the topsoil of a mining area and provides the necessary theoretical support for their management.

## 2. Materials and Methods

### 2.1. Sample Collection

**Figure 1:**
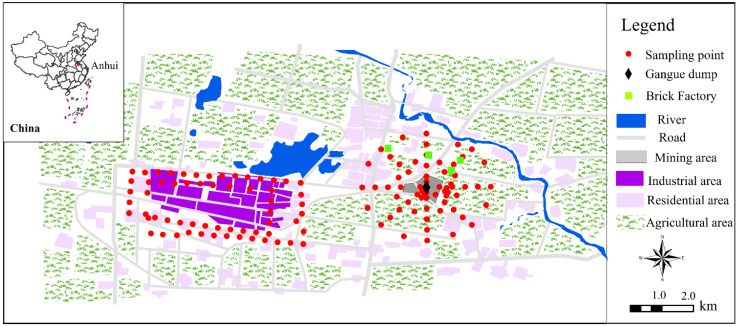
Geographical location of the research area and distribution of sampling points.

The Huaibei coalfield is an important coal mining and industrial base in China. It is located in the north of Huaibei City, Anhui Province, China (33°16′–34°14′ N; 116°23′–117°02′ E). The field has an annual coal output of more than 30 million tons and is dominated by Carboniferous and Permian coal-bearing rock series. Two sampling sites were selected within the Huaibei coalfield. The Tongting mining area was selected since it is one of the most important coal mining areas in the Huaibei coalfield and has a large coal gangue dump. There is no complex industrial activity around the mine. A circular sampling model was used from the center of the coal gangue piles with a sampling radius of 2 km (Figure 1). Sampling areas included the mining area, agricultural area, and residential area. A total of 66 samples were collected. The other site was the industrial area. The sampling points were arranged around the industrial area with 61 points, and their distribution shape was rectangular. The surface dust was swept away for sampling, and 0–10 cm soil was collected as topsoil samples with a shovel. Three parallel samples were collected within 5 m^2^ and mixed evenly to constitute one sub-sample. The samples were wrapped in aluminum foil and stored in cold storage. In addition, one coal (TTC) and two coal gangue samples were collected in the mining area, including one fresh coal gangue (TTF-1) and one weathered coal gangue (TTW-1). The sampling time was October 2020, and the geographic coordinates of the sampling points were recorded and are shown in Figure 1 and Table S1.

### 2.2. Chemicals and Sample Pretreatment

The standard included a mixture of 16 EPA-priority PAHs and APAH standard solutions. The standard mixture of 16 EPA-priority PAHs included naphthalene (NAP), acenaphthylene (ACY), acenaphthene (ACE), fluorene (FLU), phenanthrene (PHE), anthracene (ANT), fluoranthene (FLA), pyrene (PYR), benzo[a]anthracene (BaA), chrysene (CHR), benzo [b] fluoranthene (BbF), benzo [k] fluoranthene (BkF), benzo[a]pyrene (BaP), indeno [1,2,3-cd]pyrene(InP), dibenzo[a,h]anthracene (DBA), and benzo[g,h,i]perylene (BgP). APAHs included 1-methylnaphthalene (1-M-NAP), 1,2-dimethylnaphthalene (1,2-D-NAP), 2-methylanthracene (2-M-ANT), 3,6-dimethylphenanthrene (3,6-D-PHE), 1-methylpyrene (1-M-PYR), and 7-methylbenzo [a] pyrene (7-M-BaP). Deuterium-labeled standards included naphthalene-d8 (NAP-d8), acenaphthene-d10 (ACE-d10), phenanthrene-d10 (PHE-d10), chrysene-d12 (CHR-d10), and perylene-d12 (PER-d12), and were used as internal standards. Standards were purchased from AccuStandard (USA). All reagents applied were high-performance liquid chromatography (HPLC)-grade. Dichloromethane (DCM) and n-hexane (HEX) were purchased from Thermo Fisher (USA). Anhydrous sodium sulfate (Aladdin, China) was dried at 400 °C for 4 h and then placed in an oven at 60 °C before use. The pretreatment steps used have been described and verified previously [25].

### 2.3. Instrumental Analysis

The compounds were quantified using gas chromatography (Agilent 7890B)-tandem triple quadrupole mass spectrometry (Waters, Xevo TQ-GC) with full scan mode. The gas chromatograph was equipped with a DB-5MS column (length, 30 m; internal diameter, 0.25 mm; film thickness, 0.25 mm). Helium was used as the carrier gas with a constant flow rate of 1.0 mL/min. The injection mode was splitless and the injection volume was 1.0 µL. The inlet temperature was set at 280 °C. The initial oven temperature was 70 °C for 1 min, increased to 180 °C at a rate of 15 °C/min with a 2-min hold time, increased to 230 °C at a rate of 10 °C/min with a 0.5-min hold time, increased to 250 °C at a rate of 5 °C/min with a 2-min hold time, and, finally, increased to 300 °C at a rate of 8 °C/min with a 5-min hold time. Mass spectrometry was performed using an electron bombardment ionization source (electron impact: 70 eV) at an ion source temperature of 250 °C, interface temperature of 280 °C, and acquisition range of 50–550 aum, with solvent delay set to 4.0 min.

### 2.4. Quality Control

All glassware in contact with the samples was rigorously cleaned before and between use to avoid contamination. The carryover showed no detectable PACs contamination. One method blank and one sample duplicate were inserted after every 10 samples and one spiked blank was inserted after every 20 samples. The recoveries of the samples ranged from 78.5% to 128%. The range of standard deviation of the sample duplicates was 2.3–13%.

### 2.5. Qualitative and Quantitative Analyses

Target compounds were analyzed by MassLynx V4.2 (Waters, Milford, MA, USA). The qualitative analysis of PACs was completed using reference substance retention time (RT) and fragment ion data, combined with mass spectrograms in the 2017 NIST Mass Spectral Library (NIST 17) and biomarker-related books.

The internal standard method with a relative response factor was used for quantitative analysis. First, a mixed standard solution of 27 PACs (16 PAHs, seven APAHs and five internal standards) was prepared. The concentration of each component was integrated with its peak area to obtain a linear correlation coefficient. The ratio of each component’s slope (peak area/compound concentration) to the slope of the curve of the corresponding internal standard was calculated. Some target compounds without a standard were quantified according to the RF of its homolog or the PAH/APAH with the closest retention time in chromatograms. For example, monomethylnaphthalene (C1-NAP) used the RF of 1-M-NAP, whereas dimethylnaphthalene (C2-NAP), trimethylnaphthalene (C3-NAP), and tetramethylnaphthalene (C4-NAP) used the RF of 1,2-D-NAP. More isomers above C3 had similar peak times, usually with overlapping peaks. Because it was not possible to identify all alkylated isomers and accurately quantify all APAHs individually, we classified them by substituent carbon number, such as C3-NAP and C4-NAP.

### 2.6. Data Analysis

PAC data of 127 topsoil samples was statistically analyzed using Microsoft Excel (Microsoft Inc., Redmond, WA, USA) and Origin Software Version 9.0 (Origin Lab Inc., Northampton, MA, USA). The spatial distributions of PAHs were mapped using ArcGIS Ver. 10.2 (ESRI, Redlands, CA, USA) and kriging interpolation to create a continuous contour map of PAH contamination in the topsoil. The principal component analysis (PCA) was performed using SPSS Ver. 26.0 (IBM, Almonk, NY, USA), which was used to analyze the relationship among components of PACs. The cancer risk (CR) of topsoil was assessed by 16PAHs and APAHs toxicity equivalent factor (Supplementary Materials).

## 3. Results and Discussion

### 3.1. PAC Concentrations

**Figure 2:**
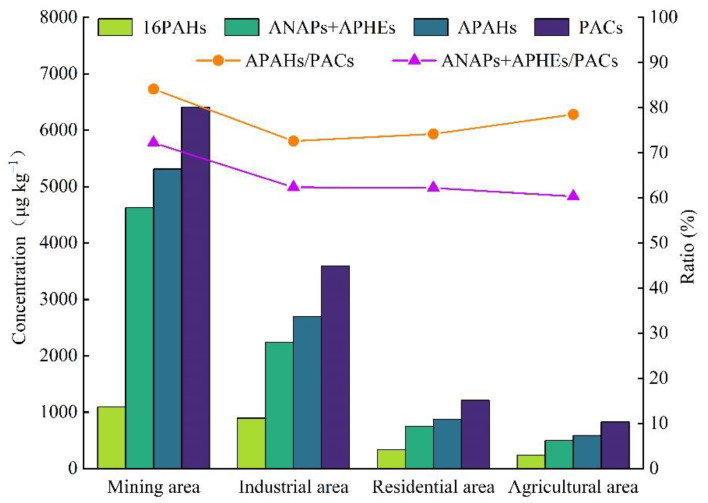
Concentration of 16 PAHs, APAHs, and PACs in topsoil and percentage content of APAHs and ANAPs + APHEs.

The topsoil samples contained not only conventional 16 PAHs, but also abundant APAHs. The APAHs with good reproducibility and selectivity include alkylnaphthalenes (ANAPs), alkylphenanthrenes (APHEs), 2-M-ANT, C1-FLU, C1-CHR, C1-FLA, C1-PYR, and 7-M-BaP. As shown in Figure 2, the average concentrations of 16 PAHs, ANAPs + APHEs, APAHs, and PACs in different functional areas showed the same trend: mining area > industrial area > residential area > agricultural area. The mining and industrial areas were the most polluted, with mean PAC concentrations of 6408 and 3592 µg/kg, respectively. The concentrations in residential and agricultural areas were 1211 and 828 µg/kg, respectively (Table 1). The mean concentrations of APAHs were significantly higher than those of 16 PAHs, accounting for 71–83% of PACs and representing the primary pollutants. Additionally, ANAPs and APHEs were the primary contributing pollutants among APAHs, accounting for 60–72% of total PACs.

There have been many studies on 16 PAHs in topsoil. We compared our results with previous studies on 16 PAHs in the topsoil of coal mining areas (previous results are shown in Table S2). Among the previous studies, the mean concentration range of 16 PAHs in the coal mining areas was260–1541 µg/kg, with the highest in Tiefa and Heshan, with 1541 and 1280 µg/kg, which are higher than that in Tongting (1095 µg/kg). Among the previous studies, the mean concentration range of 16 PAHs in residential and agricultural areas was 85–4562 µg/kg and 178–1910 µg/kg. The average concentrations of 16 PAHs in residential areas of Newcastle, Australia, and Lanzhou, China were 5358 and 954 µg/kg, respectively, higher than those in this study. The mean concentration range of 16 PAHs in the industrial areas of Nanjing, Tianjin, Lanzhou, Suzhou, and the Yangtze River Delta was 353–2240 µg/kg, while that in Newcastle, Australia, and Novocherkassk, Russia was 95,573 and 9463 µg/kg, respectively. There are few studies on APAHs in soil. Wei et al., (2015) reported that the average concentration of APAHs in Xi’an suburban surface soil was 404 µg/kg [26], whereas Chen et al., (2017) reported that the average concentration of APAHs in agricultural soil from the Yangtze River Delta was 51 µg/kg, which is considerably lower than that in the agricultural area of this study (588 µg/kg).

In this study, the concentration of PACs in coal and coal gangue was significantly higher than that in topsoil. The highest PAC concentration recorded in a coal sample was 71,367 µg/kg, and that in fresh coal gangue and weathered coal gangue was 25,815 and 14,388 µg/kg, respectively (Table 1). In those samples, APAHs accounted for 88%, 82%, and 86%, respectively, which is slightly higher than topsoil (71–83%) (Table S3). The PAC concentration in fresh coal gangue was higher than that in weathered coal gangue, possibly because the weathering process can reduce the content of PACs. For example, light, wind, and rain erosion could cause the coal gangue to fracture and reduce its particle size, gradually granulating the coal gangue. In addition, PACs gradually migrate to the surrounding environment. Moreover, through rain erosion, PACs may dissolve in the rain and eventually migrate to the groundwater and surrounding soil, posing a potential threat to the surrounding environment. Therefore, the high concentration of PACs in the surface soil around the mining area is probably related to the mining, stacking, transportation and weathering of coal and coal gangue.

### 3.2. PAC Composition Profiles in Topsoil

**Figure 3:**
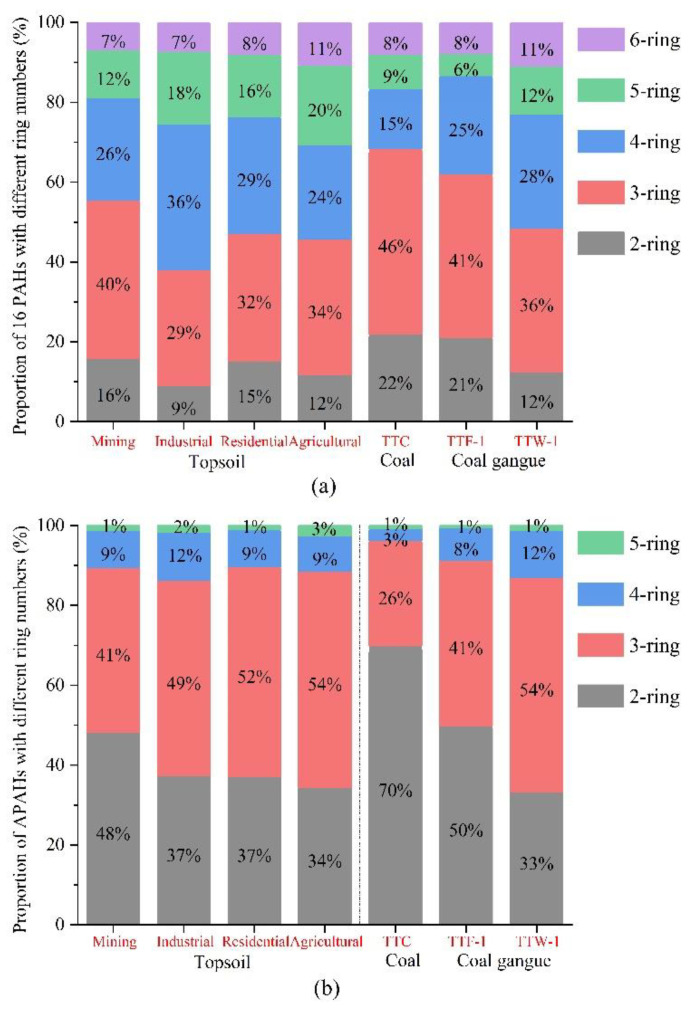
Percentage distribution of 16 PAHs (**a**) and APAHs (**b**) with different ring numbers from topsoil, coal, and coal gangue.

In the coal sample, the 16 PAHs were mainly composed of 2- and 3-rings, while in the coal gangue and topsoil, PAHs were mainly composed of 3- and 4-rings (Figure 3a). In the mining area topsoil, 2- and 3-ring 16 PAHs dominated, accounting for 56%, whereas in the topsoil of the industrial area the 4- to 6-ring dominated, accounting for 61%. The proportions of 4- to 6-ring 16 PAHs in the residential and agricultural areas were slightly higher than that in mining and industrial areas, accounting for 53% and 55%, respectively. Additionally, the proportions of 2- and 3-ring PAHs in coal and fresh coal gangue were higher than that in topsoil, accounting for 68% and 62%. The composition profiles of the 16 PAHs in the weathered coal gangue were similar to that in the topsoil of the residential area (Figure 3a).

As shown in Figure 3b, 2- and 3-ring APAHs were dominant across various sample sites, and the main contributors were ANAPs and APHEs. The proportions of 2- and3-ring APAHs in coal, fresh coal gangue, weathered coal gangue, and topsoil samples were 96%, 91%, 86%, and 89%, respectively. APAHs and 16 PAHs are abundant in coal and coal gangue [27,28]. Moreover, Laumannet et al., (2011) showed that APAHs and 16 PAHs are commonly contained in coal and coal gangue, supporting our findings [29].

In this study, the primary petrogenic sources were 2- and 3-ring PAHs, while the 4- to 6-ring PAHs produced by pyrolysis were dominant [30]. Coal and coal gangue are also petrogenic sources. The PAC composition pattern of topsoil in the mining area was similar to that of coal and coal gangue, indicating that topsoil pollution was affected by coal and coal gangue. This is likely linked to coal mining and transportation, which produces scattered coal ash and coal blocks, and the large coal gangue dump in the mining area. Therefore, as with coal and coal gangue, the topsoil PACs in mining area were mainly 2- and 3-rings. Additionally, the proportion of 4- to 6-ring PAHs in the industrial area topsoil was higher than that of other functional areas, which is probably related to the numerous coal-burning activities that occur.

### 3.3. PAC Spatial Distributions in Topsoil

**Figure 4:**
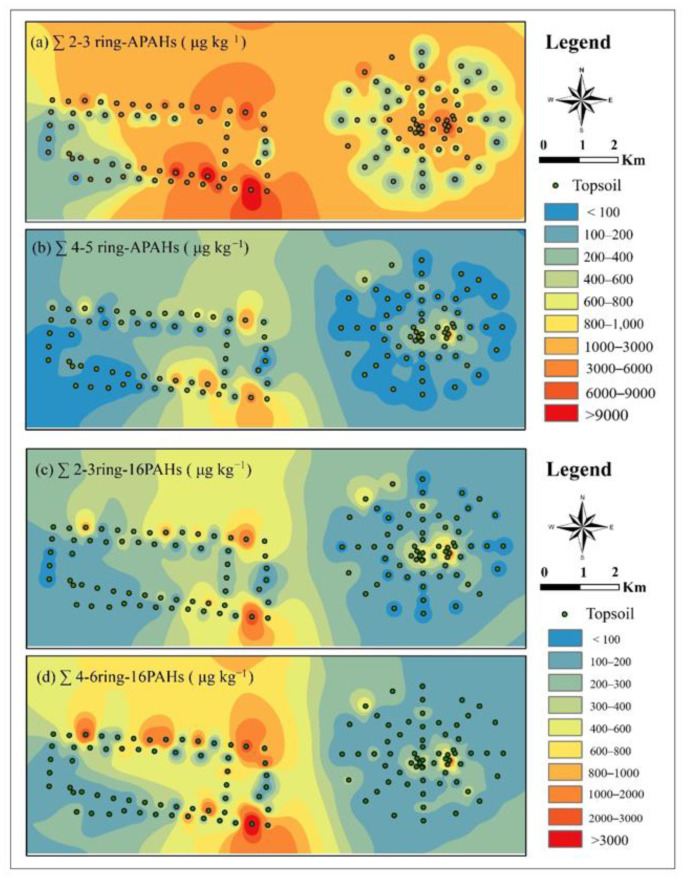
Spatial distribution of ∑2- and 3-ring-APAHs (**a**), ∑4- and 5- ring-APAHs (**b**), ∑2- and 3-ring-16 PAHs (**c**), and ∑4- to 6-ring-APAHs (**d**) in topsoil samples.

The spatial distributions of ∑2- and 3-ring-APACs, ∑4- and 5-ring-APAHs, ∑2- and 3-ring-16PAHs, ∑4- and 5-ring-16PAHs, ∑APAHs, and ∑16PAHs were mapped using ArcGIS based on the inverse distance weighted spatial interpolation method (Figure 4, and Figure S1). The coal mining areas and the eastern part of the industrial park were heavily polluted, and the pollution level of APAHs was significantly higher than that of 16 PAHs (Figure S1). The spatial distribution pattern of APAHs and 16 PAHs with different ring numbers were consistent (Figure 4). The correlation coefficients (R^2^) of 16 PAHs and APAHs in mining, industrial, agricultural, and residential areas were 0.93, 0.86, 0.83, and 0.64, respectively (Figure S2), suggesting that they are from the same source, that is, coal mining and industrial production.

PAC concentrations in the mining area were significantly higher than that in surrounding agricultural and residential areas, which may be related to coal and gangue piling and transportation in the mining area. Additionally, the PAC pollution in the topsoil near a brick factory was heavier than that in other areas of the agricultural area. This may be due to a large amount of coal and coal gangue in the brick factory and the daily coal burning to power production activities, both PAC pollution sources. Easterly winds dominate the study area; thus, PACs released by coal burning in the brick factories settle on the residential areas in the west. Therefore, the PAC pollution of the residential area west of the brick factory is heavier than in residential areas in the southeast. The results of this study can provide data support for pollution prevention and control around coal mine areas.

The PAC pollution in the industrial area that depends on coal was more significant, showing the distribution characteristics of high pollution in the east and low pollution in the west. This may be related to the distribution of industry types. In the eastern part of the industrial area, there are mainly coal preparation sites, power plants, cement plants, and coking plants. In these sites, there are significant amounts of coal and coal gangue, and widespread industrial coal burning occurs, contributing to the release of PAC pollutants. The west is dominated by biotechnology and materials technology, with little industrial activity. Moreover, as mentioned above, the region is dominated by easterly winds. Therefore, the spatial distribution of PAC pollution has a trend of stepwise decline from the east to the west.

### 3.4. Identification of the Source of PACs

#### 3.4.1. Principal Component Analysis (PCA)

**Figure 5:**
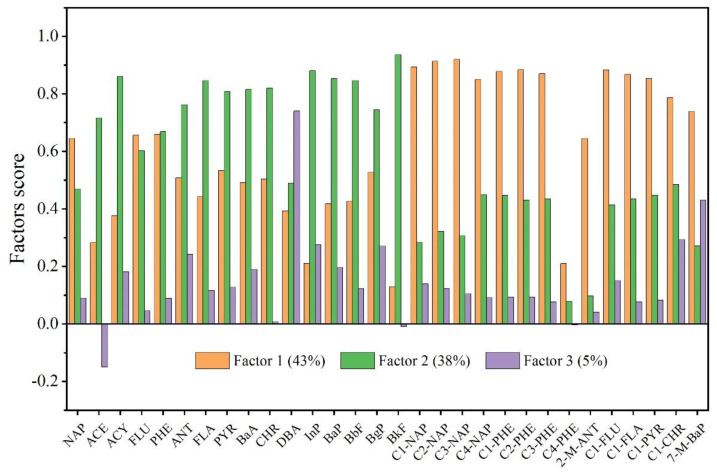
Factor score of principal component analysis.

Principal component analysis is a multivariate analytical tool widely used for receptor modeling in soil source apportionment studies. [10,31,32]. In this paper, PCA was conducted on 136 topsoil data to extract 3 factors with eigenvalues greater than 1, and the sum of their variance contribution rates was 86% (Figure 5). The variance contribution rate of factor 1 was 43%, and C1-C4NAP, C1-C3PHE, and C1-FLU had higher loadings. Their correlation coefficients (R^2^) ranged from 0.862 to 0.993 (Table S4). As for the 16 PAHs, NAP, FLU, and PHE had higher loads and had R^2^ values of 0.916, 0.904 and 0.967, respectively. Usually, 2- to 3-ring PAHs are mainly derived from petrogenic sources [33,34]. 1-M-NAP and 2-M-NAP are associated with petrogenic sources [35,36]. APAHs of different coal ranks are mainly 2- to 3-rings [29], and coal is one of the petrogenic sources. Therefore, factor 1 was identified as a petrogenic source. The variance contribution rate of factor 2 was 38%. The high-loading compounds were ACY, FLA, CHR, BbF, BkF, BaP, and InP, and they all had a good correlation (Table S4). High-temperature combustion mainly produces 4- to 6-ring PAHs [30,37]. The high contribution of FLA is related to coal and wood (biomass) burning [38,39]. CHR, BbF, and BkF can be used as tracers for residential coal combustion [34,36,40,41]. In addition, BbF, BkF, BaP, and InP are usually released through biomass burning [36,37,42], and ACY is released through wood burning [43]. Therefore, factor 2 was identified as coal and biomass combustion. The variance contribution rate of factor 3 was 5%; DBA has the highest load, followed by 7-M-BaP, C1-CHR, and BgP. CHR, DBA, BaP, and BgP are common indicators of vehicle exhaust [27,43,44]. CHR represents diesel vehicle emissions [45]. Therefore, factor 3 was identified as vehicle emissions.

#### 3.4.2. Fingerprinting Information Comparison

**Figure 6:**
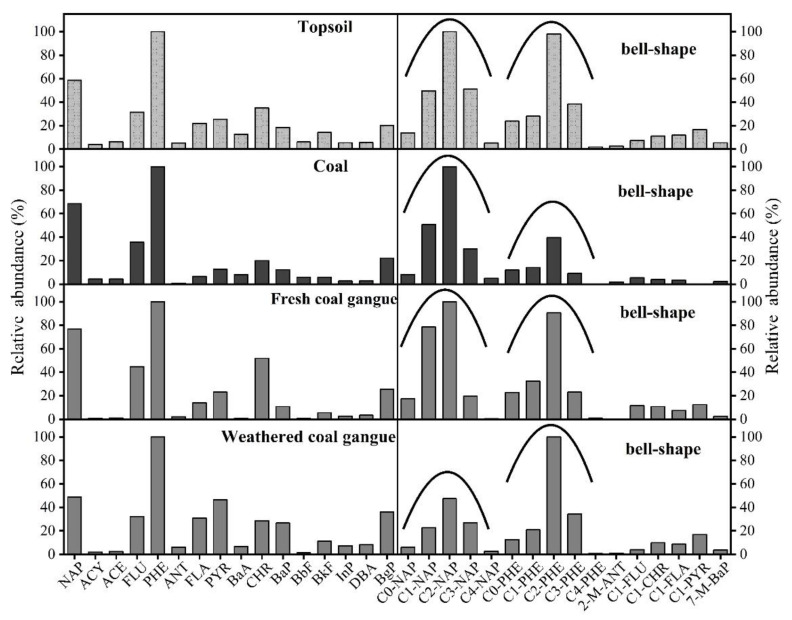
PAC fingerprint information and bell-shaped distribution pattern from different types of samples. The bell-shaped distribution means that the content of APAHs is higher than that of parent PAHs, and the relative abundance of C0–C4 PAHs is high in the middle and low on both sides.

As shown in Figure 6, the fingerprint information of soil and coal, fresh coal gangue and weathered coal gangue in the mining area have a high matching degree, indicating that topsoil PACs probably came from coal and coal gangue. In addition, the relative abundance of C1–C4 NAP and C1–C4 PHE in all samples showed a “bell-shaped” distribution. Generally, the pattern of C1–C4 APAHs from a petrogenic origin is “bell-shaped” [46,47]. Currently, petroleum is considered to be the main source of APAHs in the environment, namely, the petrogenic source [48]. However, coal and coal gangue in mining areas are also a petrogenic source but have been neglected in the literature.

China is the world’s largest producer and consumer of coal. In China, 75 percent of primary energy comes from coal burning, and will likely continue to dominate for the next 50 years [49,50]. Coal and its by-product coal gangue contain many organic and inorganic substances [18,28,51,52]. In the long-term coal mining process of the Huaibei coalfield, PACs may be released through coal washing wastewater, coal ash, and coal overflow during vehicle transportation. Additionally, there is considerable coal gangue accumulation in the production process. Coal burning activities also exist in and around the mining area. Long-term stacking and burning of coal and gangue produce harmful substances such as PACs, which pollute the surrounding environment and cause potential risks to human health [53,54,55]. In addition, coal gangue is gradually fragmented after long-term weathering, and PACs in it gradually escape to the surrounding environment and eventually settle and migrate through the soil by rain leaching [56,57]. The farmland soil inside and outside the mining area is polluted to varying degrees. These findings highlight that the typically neglected petrogenic sources, coal and coal gangue, required greater attention as PAC sources than before.

### 3.5. Health Risk Assessment

The toxic equivalent quantity of Bap (TEQ_BaP_) concentration of each PAC component is the PAH concentration multiplied by its toxicity equivalent factor (TEF) value (Table S5). In the calculation of TEQ_BaP_ of APAHs, the TEF corresponding to parent PAHs was used instead. APAHs are more toxic than parent PAHs [5,6]. If the TEQ_BaP_ of only 16 PAHs is calculated, the actual TEQ_BaP_ is underestimated by at least 13–35% (Table S5). In this paper, three exposure methods were used to calculate the carcinogenic risk value of PACs (CRs; the calculation formula is shown in the Supplementary Materials). The parameters used for the incremental life cycle cancer risk assessment are shown in Table S6. The USEPA cancer risk assessment is divided into three levels: negligible risk of cancer (<10^−6^), potential risk of cancer (10^−6^–10^−4^), and high risk of cancer (>10^−4^) [58,59]. In general, CRs of different functional areas show a trend of mining area > industrial area > residential area > agricultural area. All CRs were lower than 10^−4^, indicating no high carcinogenic risk in the study area. The cancer risk from dermal contact was highest, whereas the inhalation pathway was lowest. In the mining and industrial areas, the maximum values of children’s CR_derm_ were 1.36 × 10^−5^ and 1.60 × 10^−5^, indicating a potential carcinogenic risk. CRs of adults were all less than 10^−6^, which is within the range of negligible risk (Table 2). However, PACs are multi-source pollutants, and their cancer risk may continue to increase if emissions are not controlled at the source in the future.

## 4. Conclusions

APAHs generally exist in the topsoil of different functional areas in the Huaibei coalfield and are the main contributors to PACs. The mean concentration of APAHs (4734 µg/kg) accounted for 76% of PACs (6255 µg/kg). The mean PAC concentration in different functional areas was significantly different, showing a trend of mining area (6408 µg/kg)> industrial area (3592 µg/kg) > residential area (1211 µg/kg) > agricultural area (828 µg/kg). The average concentration of APAHs and 16 PAHs showed the same trend. The 16 PAHs and APAHs were mainly of low molecular weight (under 4-rings) and had significant diagenetic source characteristics. The spatial distribution pattern of PACs with different ring numbers was consistent, indicating that they are from the same source. Source identification showed that the 43% contribution rate of PACs in the topsoil came from a petrogenic source. By comparing the fingerprint information of coal and coal gangue, we inferred that coal and coal gangue was the primary petrogenic source. Coal ash, coal overflow during vehicle transportation, coal burning activities, and coal gangue dump weathering are mainly the anthropogenic influence of coal utilization. There are potential cancer hazards in some parts of the mining area. However, there is a lack of research on the toxicity equivalent factors (TEFs) of APAHs. Extensive ecological risk criteria for assessing individual APAHs should be developed. Coal fields similar to our study area are widely distributed; therefore, investigating the widespread pollution of PACs derived from coal mining areas deserves further attention.

## Figures and Tables

**Table 1:** PAC concentrations (µg/kg) in topsoil from different functional groups and coal and coal gangue.

Compounds	Mining Area Topsoil (*n* = 15)			Industrial Area Topsoil (*n* = 61)			Residential Area Topsoil (*n* = 6)			Agricultural Area Topsoil (*n* = 45)			Mining Area Coal (*n* = 1)	Mining Area Coal Gangue (*n* = 2)	
	Min	Max	Mean	Min	Max	Mean	Min	Max	Mean	Min	Max	Mean	TTC	TTF-1	TTW-1
NAP	54	392	173	20	421	81	nd	143	51	nd	152	28	1927	969	255
ACY	nd	39	12	nd	77	13	nd	nd	10	nd	nd	10	123	10	10
ACE	nd	57	18	nd	131	20	nd	nd	13	nd	25	13	123	13	13
FLU	29	266	93	nd	419	43	nd	49	20	nd	88	16	1004	564	167
PHE	92	797	296	nd	1785	170	nd	140	60	nd	288	36	2810	1263	520
ANT	nd	83	15	nd	209	14	nd	nd	6	nd	13	6	23	27	31
FLA	nd	357	65	nd	1068	108	nd	70	30	nd	113	16	184	177	160
PYR	nd	372	75	nd	840	89	nd	71	26	nd	81	15	358	292	242
BaA	nd	176	37	nd	349	33	nd	19	11	nd	37	11	224	10	35
CHR	14	347	104	nd	1044	97	nd	92	31	nd	107	15	563	655	148
BbF	nd	179	55	nd	995	73	nd	22	10	nd	79	10	163	8	8
BkF	nd	61	18	nd	215	30	nd	31	18	nd	33	15	165	71	58
BaP	nd	249	42	nd	505	47	nd	23	13	nd	25	11	345	137	139
InP	nd	62	16	nd	125	19	nd	13	13	nd	13	13	74	33	38
DBA	nd	45	16	nd	47	13	nd	12	12	nd	12	12	82	43	43
BgP	nd	240	60	nd	528	46	nd	25	15	nd	13	13	625	322	188
ANAPs	659	7240	2561	26	9838	1011	26	1016	324	26	2011	203	43,711	10,583	4093
APHEs	490	6213	2065	52	12,669	1229	62	1415	429	60	2190	297	14,864	8138	6411
2-M-ANT	nd	75	33	nd	527	36	nd	26	12	nd	44	11	409	8	44
C1-FLU	nd	333	93	nd	581	48	nd	53	17	nd	74	10	1257	642	168
C1-FLA	25	448	147	nd	902	104	nd	74	24	nd	130	14	771	421	356
C1-FLA	25	448	147	nd	902	104	nd	74	24	nd	130	14	771	421	356
C1-PYR	nd	789	207	nd	1531	158	nd	156	43	nd	228	27	63	692	696
7-M-BaP	nd	516	68	nd	456	47	nd	22	10	nd	88	16	551	136	155
∑16PAHs	307	3721	1095	152	8758	896	152	735	338	151	1088	239	8793	4594	2055
∑APAHs	1233	16,270	5313	114	27,380	2696	129	2794	872	127	4886	588	62,575	21,221	12,329
∑PACs	1541	19,991	6408	266	36,138	3735	281	3529	1252	278	5974	847	71,368	25,815	14,388

nd: not detected.

**Table 2:** Cancer risks for local children and adults on exposure to ∑ (16APHs + APAHs) via various pathways.

Location	CRs	Child			Adult		
		Mean	Max	Min	Mean	Max	Min
Mining	CR_ing_^a^	8.14 × 10^−7^	4.06 × 10^−6^	3.21 × 10^−7^	6.96 × 10^−8^	2.64 × 10^−7^	3.71 × 10^−8^
	CR_derm_ ^b^	2.73 × 10^−6^	1.36 × 10^−5^	1.08 × 10^−6^	4.76 × 10^−7^	1.80 × 10^−6^	2.54 × 10^−7^
	CR_inh_^c^	1.20 × 10^−11^	5.98 × 10^−11^	4.72 × 10^−12^	2.70 × 10^−11^	1.02 × 10^−10^	1.44 × 10^−11^
	CR_total_	3.55 × 10^−6^	1.77 × 10^−5^	1.40 × 10^−6^	5.45 × 10^−7^	2.07 × 10^−6^	2.91 × 10^−7^
Industrial	CR_ing_	6.91 × 10^−7^	4.78 × 10^−6^	3.08 × 10^−7^	9.05 × 10^−8^	6.26 × 10^−7^	4.04 × 10^−8^
	CR_derm_	2.32 × 10^−6^	1.60 × 10^−5^	1.03 × 10^−6^	6.19 × 10^−7^	4.28 × 10^−6^	2.76 × 10^−7^
	CR_inh_	1.02 × 10^−11^	7.04 × 10^−11^	4.54 × 10^−12^	3.51 × 10^−11^	2.43 × 10^−10^	1.56 × 10^−11^
	CR_total_	3.01 × 10^−6^	2.08 × 10^−5^	1.34 × 10^−6^	7.09 × 10^−7^	4.90 × 10^−6^	3.16 × 10^−7^
Residential	CR_ing_	3.32 × 10^−7^	4.31 × 10^−7^	3.08 × 10^−7^	4.36 × 10^−8^	5.65 × 10^−8^	4.04 × 10^−8^
	CR_derm_	1.12 × 10^−6^	1.45 × 10^−6^	1.03 × 10^−6^	2.98 × 10^−7^	3.86 × 10^−7^	2.76 × 10^−7^
	CR_inh_	4.90 × 10^−12^	6.35 × 10^−12^	4.50 × 10^−12^	1.69 × 10^−11^	2.19 × 10^−11^	1.57 × 10^−11^
	CR_total_	1.45 × 10^−6^	1.88 × 10^−6^	1.34 × 10^−6^	3.41 × 10^−7^	4.43 × 10^−7^	3.16 × 10^−7^
Agricultural	CR_ing_	3.46 × 10^−7^	7.33 × 10^−7^	3.08 × 10^−7^	4.54 × 10^−8^	9.61 × 10^−8^	4.03 × 10^−8^
	CR_derm_	1.16 × 10^−6^	2.46 × 10^−6^	1.03 × 10^−6^	3.10 × 10^−7^	6.57 × 10^−7^	2.76 × 10^−7^
	CR_inh_	5.10 × 10^−12^	1.08 × 10^−11^	4.54 × 10^−12^	1.76 × 10^−11^	3.73 × 10^−11^	1.56 × 10^−11^
	CR_total_	1.51 × 10^−6^	3.19 × 10^−6^	1.34 × 10^−6^	3.55 × 10^−7^	7.53 × 10^−7^	3.16 × 10^−7^

^a^ Cancer risk through ingestion of soil particles. ^b^ Cancer risk from dermal contact. ^c^ Cancer risk from inhalation.

## Data Availability

The datasets used or analyzed during the current study are available from the corresponding author on reasonable request.

## Supplementary Materials

The following supporting information can be downloaded at: https://www.mdpi.com/article/10.3390/ijerph191912733/s1, Table S1: The geographic coordinates and sampling areas of sampling points. Table S2: Topsoil PAC concentrations from this study compared with previous studies (µg/kg). Table S3: Concentrations and proportions of PACs components in topsoil, coal and coal gangue (µg/kg). Figure S1 Spatial distribution of ∑16 PAHs (a), and ∑APAHs (b) in topsoil samples. Figure S2: Correlation analysis of APAHs and 16PAHs in topsoil. Table S4: The correlation coefficients between the PAC components. Table S5: TEQ_BaP_ of 16 PAHs and APAHs in topsoil of different functional areas in Huaibei Coalfield. Table S6: Parameters used in the incremental lifetime cancer risk assessment. Some references are cited in the Supplementary Materials [7,18,22,26,57,60,61,62,63,64,65,66,67,68,69,70,71,72,73,74,75,76,77,78,79,80,81,82,83,84].
