# Supplementary material for: Alkylated Polycyclic Aromatic Hydrocarbons Are the Largest Contributor to Polycyclic Aromatic Compound Concentrations in the Topsoil of Huaibei Coalfield, China
Source: Int J Environ Res Public Health. 2022 Oct 5;19(19):12733. doi: 10.3390/ijerph191912733 (PMC9566202; doi:10.3390/ijerph191912733)
Supplement: Supplementary file 1 [file ijerph-19-12733-s001.zip › ijerph-1892665-supplementary.pdf]

Table S1 The geographic coordinates and sampling areas of sampling points.

Sampling points	Longitude	Latitude	Sampling areas	Sampling sites	Longitude	Latitude	Sampling areas
Gangue dump	116.65827155	33.61358732	Mining area	TT-8-4	116.66716576	33.59760583	Residential area
TT-1-2	116.66501626	33.61326003	Mining area	TT-8-7	116.63875580	33.62140944	Residential area
TT-1-3	116.66543560	33.61177262	Mining area	TT-8-8	116.65025711	33.63027157	Residential area
TT-1-4	116.66494610	33.61069913	Mining area	ID-1-1	33.61523362	116.56493349	Industrial area
TT-1-5	116.65828228	33.61181373	Mining area	ID-1-2	33.61514641	116.56923376	Industrial area
TT-1-6	116.65681243	33.61231409	Mining area	ID-1-3	33.61476503	116.57354031	Industrial area
TT-1-7	116.65611506	33.61359179	Mining area	ID-1-4	33.61490063	116.57781574	Industrial area
TT-1-8	116.66259623	33.61357734	Mining area	ID-1-5	33.61468836	116.58221540	Industrial area
TT-1-9	116.66445147	33.61219983	Mining area	ID-1-6	33.61419328	116.58647136	Industrial area
TT-2-2	116.66654912	33.61451866	Mining area	ID-1-7	33.61387706	116.59212789	Industrial area
TT-2-3	116.66259527	33.61360966	Mining area	ID-1-8	33.61307048	116.59895021	Industrial area
TT-2-5	116.65831447	33.60999543	Mining area	ID-1-9	33.61240903	116.60625808	Industrial area
TT-2-6	116.65524602	33.61106318	Mining area	ID-1-10	33.60882475	116.60604308	Industrial area
TT-2-7	116.65751417	33.61105661	Mining area	ID-1-11	33.60519561	116.60578515	Industrial area
TT-2-8	116.6581163	33.60988229	Mining area	ID-1-12	33.60160207	116.60550576	Industrial area
TT-2-9	116.6565999	33.61006732	Mining area	ID-1-13	33.59786087	116.60531759	Industrial area
TT-1-1	116.65828228	33.61538768	Agricultural area	ID-1-14	33.59836587	116.60096065	Industrial area
TT-2-1	116.65826619	33.61718353	Agricultural area	ID-1-15	33.59885381	116.59670129	Industrial area
TT-2-4	116.66131854	33.61103638	Agricultural area	ID-1-16	33.59935599	116.59238394	Industrial area
TT-3-1	116.65827692	33.62077959	Agricultural area	ID-1-17	33.59986342	116.58815340	Industrial area
TT-3-2	116.66438699	33.61865771	Agricultural area	ID-1-18	33.60053704	116.58391851	Industrial area
TT-3-3	116.66691899	33.61363646	Agricultural area	ID-1-19	33.60110418	116.57968459	Industrial area
TT-3-4	116.66438699	33.60848536	Agricultural area	ID-1-20	33.60162289	116.57550523	Industrial area
TT-3-5	116.65836275	33.60640339	Agricultural area	ID-1-21	33.60246434	116.57119827	Industrial area
TT-3-6	116.65215611	33.60850770	Agricultural area	ID-1-22	33.60332429	116.56699404	Industrial area
TT-3-7	116.64964288	33.61351584	Agricultural area	ID-1-23	33.60318204	116.56476974	Industrial area

Continued

Sampling sites	Longitude	Latitude	Sampling areas	Sampling sites	Longitude	Latitude	Sampling areas
TT-3-8	116.65220976	33.61864878	Agricultural area	ID-1-24	33.60445792	116.56389906	Industrial area
TT-4-1	116.65826082	33.62436656	Agricultural area	ID-1-25	33.61165472	116.56447802	Industrial area
TT-4-2	116.66741788	33.62121289	Agricultural area	ID-2-1	33.61876925	116.55942460	Industrial area
TT-4-3	116.67122662	33.61365433	Agricultural area	ID-2-2	33.61844879	116.56368619	Industrial area
TT-4-4	116.66740179	33.60596554	Agricultural area	ID-2-3	33.61866508	116.56808797	Industrial area
TT-4-5	116.65841103	33.60278886	Agricultural area	ID-2-4	33.61828356	116.57235141	Industrial area
TT-4-6	116.64913058	33.60597895	Agricultural area	ID-2-5	33.61792065	116.57664796	Industrial area
TT-4-7	116.64532185	33.61348904	Agricultural area	ID-2-6	33.61760327	116.58091334	Industrial area
TT-4-8	116.64915204	33.62122183	Agricultural area	ID-2-7	33.61722409	116.58524405	Industrial area
TT-5-1	116.65825546	33.62796679	Agricultural area	ID-2-8	33.61732840	116.58953294	Industrial area
TT-5-2	116.67047024	33.62374567	Agricultural area	ID-2-9	33.61734410	116.59397301	Industrial area
TT-5-3	116.67552888	33.61368560	Agricultural area	ID-2-10	33.61689599	116.59817809	Industrial area
TT-5-5	116.65845394	33.59920098	Agricultural area	ID-2-11	33.61635035	116.60248071	Industrial area
TT-5-6	116.64607286	33.60338757	Agricultural area	ID-2-12	33.61590386	116.60693461	Industrial area
TT-5-7	116.64103031	33.61347564	Agricultural area	ID-2-13	33.61551172	116.61105675	Industrial area
TT-5-8	116.64607286	33.62373227	Agricultural area	ID-2-14	33.61498666	116.61597439	Industrial area
TT-6-1	116.65824473	33.63156687	Agricultural area	ID-2-15	33.61100487	116.61615120	Industrial area
TT-6-2	116.67351723	33.62628730	Agricultural area	ID-2-16	33.60779170	116.61646770	Industrial area
TT-6-3	116.67985260	33.61367220	Agricultural area	ID-2-17	33.60461840	116.61640834	Industrial area
TT-6-5	116.65850222	33.59563084	Agricultural area	ID-2-18	33.60209656	116.61452352	Industrial area
TT-6-6	116.64301515	33.60090334	Agricultural area	ID-2-19	33.59861154	116.61697158	Industrial area
TT-6-8	116.64302051	33.62628283	Agricultural area	ID-2-20	33.59442762	116.61685306	Industrial area
TT-7-1	116.66261673	33.62183828	Agricultural area	ID-2-21	33.59479645	116.61256328	Industrial area
TT-7-2	116.66789532	33.61747837	Agricultural area	ID-2-22	33.59504533	116.60823149	Industrial area
TT-7-3	116.66845322	33.61079513	Agricultural area	ID-2-23	33.59567050	116.60397584	Industrial area
TT-7-4	116.66287422	33.60561258	Agricultural area	ID-2-24	33.59626069	116.59971043	Industrial area

Continued

Sampling sites	Longitude	Latitude	Sampling areas	Sampling sites	Longitude	Latitude	Sampling areas
TT-7-4	116.66287422	33.60561258	Agricultural area	ID-2-24	33.59626069	116.59971043	Industrial area
TT-7-5	116.65420532	33.60536238	Agricultural area	ID-2-25	33.59679357	116.59546209	Industrial area
TT-7-6	116.64828300	33.61033050	Agricultural area	ID-2-26	33.59709487	116.59112356	Industrial area
TT-7-7	116.6488903	33.61678467	Agricultural area	ID-2-27	33.59731205	116.58683968	Industrial area
TT-7-8	116.65454865	33.62208843	Agricultural area	ID-2-28	33.59779376	116.58258900	Industrial area
TT-8-1	116.6688415	33.62787501	Agricultural area	ID-2-29	33.59829859	116.57836075	Industrial area
TT-8-2	116.67772293	33.62190975	Agricultural area	ID-2-30	33.59736460	116.57438512	Industrial area
TT-8-3	116.67832375	33.60754267	Agricultural area	ID-2-31	33.59760351	116.56963917	Industrial area
TT-8-5	116.65068626	33.59703389	Agricultural area	ID-2-32	33.59798516	116.56565478	Industrial area
TT-8-6	116.63849831	33.60654189	Agricultural area	ID-2-33	33.60482785	116.55830985	Industrial area
TT-5-4	116.67046487	33.60341437	Residential area	ID-2-34	33.60844783	116.55841725	Industrial area
TT-6-4	116.67352796	33.60087207	Residential area	ID-2-35	33.61202262	116.55869594	Industrial area
TT-6-7	116.63670391	33.61343990	Residential area	ID-2-36	33.61559712	116.55904958	Industrial area

Table S2 Topsoil PAC concentrations from this study compared with previous studies (μg kg^{-1}).

Compounds	Study location	Sample types	Mean concentration	Range	Reference
16 PAHs	Tiefa, China	Coal mining area soils	1541.1	53.6-5642.3	61
16 PAHs	Heshan, China	Coal mining area soils	1280.12	79.56-4256.96	18
16 PAHs	Yangquan, China	Coal mining area soils	259.96	49.82-702.2	58
16 PAHs	Luling, Liuer and Zhangji, China	Coal mining area soils	840	130-3540	22
16 PAHs	Tongting, China	Coal mining area dust	1710	390-3083	This study
16 PAHs	South Korea	Agricultural soils	236	23.3–2834	62
16 PAHs	Nanjing, China	Agricultural soils	178	21.91–533.84	63
16 PAHs	Delhi, India	Agricultural soils	1910	830–3880	64
16 PAHs	Guangdong, China	Agricultural soils	318	22.1–1256.9	65
16 PAHs	Tiefa, China	Agricultural soils	494.8	5.1-1249.2	61
16 PAHs	Poland	Agricultural soils	252	73-1800	66
16 PAHs	Shanghai, China	Agricultural soils	339	17.2-3775	67
16 PAHs	Tongting, China	Agricultural topsoil	239	152-1073	This study
16 PAHs	Nanjing, China	Coking plant soil	385.2	-	68
16 PAHs	Tianjin, China	Industrial soils	814	68.7-5590	69
13 PAHs	Newcastle, Australia	Industrial soils	95573	2509-392932	70
16 PAHs	Yangtze River Delta, China	Industrial soils	616	15.8–4449	71
16 PAHs	Lanzhou, China	Industrial soils	2240	391-10900	72
16 PAHs	Suzhou, China	Industrial soils	352.94	125.99-796.65	73
16 PAHs	Novocherkassk, Russia	Electric Power Station soil	9463	-	74
16 PAHs	Huaibei, China	Industrial topsoil	896	174-8704	This study
16 PAHs	Orlando, US	Urban soils	3227	43–30428	75

Continued

Compounds	Study location	Sample types	Mean concentration	Range	Reference
16 PAHs	Tampa, US	Urban soils	4562	59–58640	75
16 PAHs	Southern Italy	Urban soils	84.85	7.62-755	76
16 PAHs	Naples, Italy	Urban soils	206	nd-4191	77
16 PAHs	Clay county, US	Urban soils	1821	797–7909	78
16 PAHs	Ocala, US	Urban soils	2748	950–11,451	78
16 PAHs	Pensacola, US	Urban soils	3115	922–17698	78
16 PAHs	West Palm Beach, US	Urban soils	4055	1133–30691	78
16 PAHs	Beijing, China	Urban soils	3917	219–27825	79
16 PAHs	Shanghai, China	Urban soils	3290	442–17900	80
13 PAHs	Kumasi Metropolis, Ghana	Urban soils	442.5	14.78-2084	81
15 PAHs	Ibadan, Nigeria	Urban soils	2211	489-5616	82
15 PAHs	Valparaiso, Santiago and Punta Arenas, Chile	Urban soils	618	21.5 - 4370	81
16 PAHs	Tianjin, China	urban and rural soil	818	199-5190	83
14 PAHs	Dalian, China	urban and rural soil	1104	219-18727	84
13 PAHs	Newcastle, Australia	Residential soils	5358	201-11176	70
16 PAHs	Lanzhou, China	Residential soils	954	350-2450	72
6 APAHs	Xi’an, China	suburban soil	404	232-819	26
18 APAHs	YRD, China	agricultural soil	51	5.5-696.2	7

Table S3 Concentrations and proportions of PACs components in topsoil, coal and coal gangue (μg kg^{-1}).

Sample type	16PAHs	ANAPs+APHEs	APAHs	PACs	APAHs/PACs	(ANAPs+APHEs) /APAHs	(ANAPs+APHEs) /PACs
Mining topsoil	1095	4626	5313	6408	82.9	87.1	72.2
Industrial topsoil	896	2240	2696	3592	75.1	83.1	62.4
Residential topsoil	338	753	872	1211	72	86.3	62.2
Agricultural topsoil	239	499	588	828	71.1	84.9	60.3
coal	8791	58575	62576	71367	87.7	93.6	82.1
fresh coal gangue	4594	18721	21221	25815	82.2	88.2	72.5
weathered coal gangue	2057	10504	12331	14388	85.7	85.2	73.0

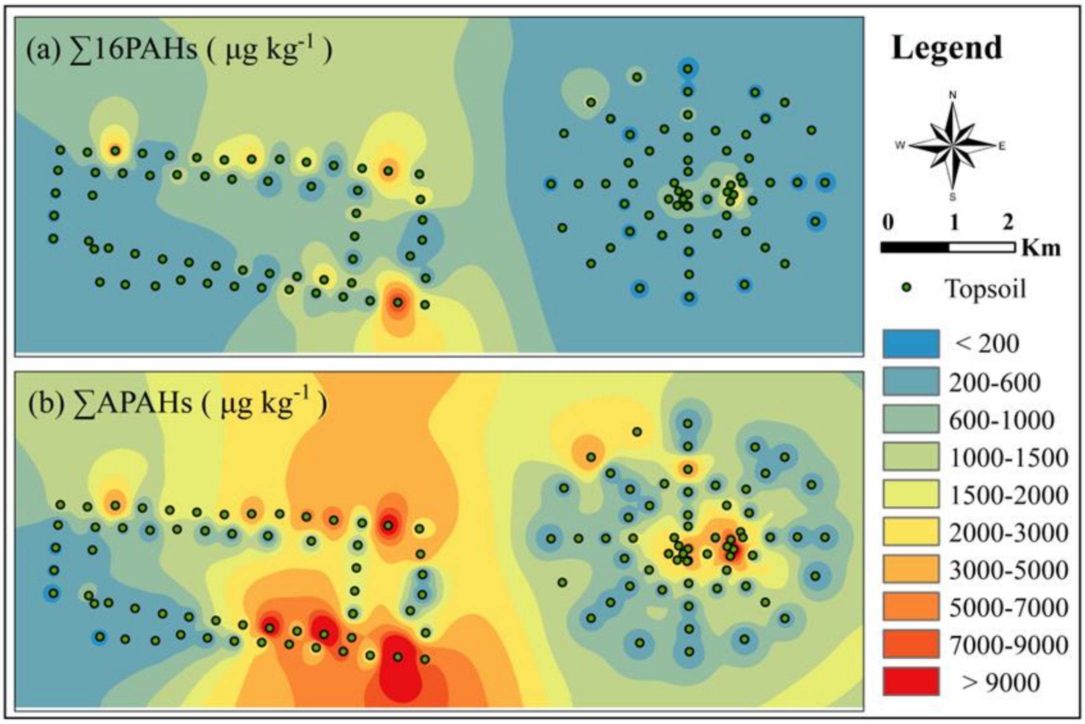

Figure S1 Spatial distribution of ∑16 PAHs (a), and ∑APAHs (b) in topsoil samples.

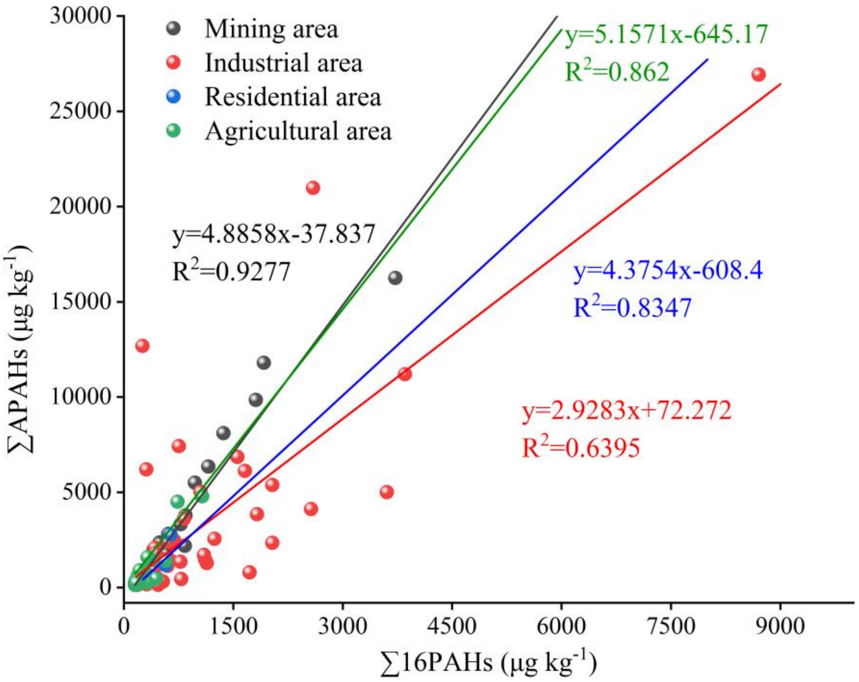

Figure S2 Correlation analysis of APAHs and 16PAHs in topsoil.

Table S4 The correlation coefficients between the PAC components.

Compounds	NAP	ACY	ACE	FLU	PHE	ANT	FLA	PYR	BaA	CHR	BbF	BkF	BaP	InP	DBA	BgP
NAP	1															
ACY	.623**	1														
ACE	.602**	.754**	1													
FLU	.916**	.759**	.691**	1												
PHE	.904**	.807**	.643**	.967**	1											
ANT	.642**	.920**	.571**	.783**	.856**	1										
FLA	.717**	.876**	.701**	.798**	.880**	.882**	1									
PYR	.789**	.889**	.709**	.874**	.939**	.900**	.978**	1								
BaA	.758**	.914**	.663**	.848**	.908**	.912**	.932**	.952**	1							
CHR	.785**	.869**	.724**	.876**	.931**	.858**	.938**	.959**	.925**	1						
BbF	.694**	.867**	.647**	.789**	.880**	.896**	.930**	.931**	.914**	.913**	1					
BkF	.554**	.869**	.791**	.641**	.691**	.735**	.848**	.812**	.825**	.817**	.833**	1				
BaP	.728**	.902**	.721**	.805**	.873**	.876**	.926**	.944**	.945**	.932**	.944**	.854**	1			
InP	.575**	.919**	.637**	.693**	.751**	.841**	.853**	.844**	.887**	.827**	.823**	.851**	.866**	1		
DBA	.570**	.675**	.360**	.608**	.669**	.735**	.673**	.690**	.717**	.604**	.697**	.508**	.727**	.702**	1	
BgP	.758**	.850**	.594**	.834**	.909**	.874**	.892**	.934**	.926**	.909**	.917**	.723**	.937**	.827**	.771**	1
C1-NAP	.867**	.608**	.514**	.846**	.844**	.664**	.651**	.732**	.717**	.702**	.633**	.424**	.661**	.507**	.602**	.726**
C2-NAP	.841**	.648**	.520**	.855**	.864**	.716**	.689**	.768**	.751**	.736**	.673**	.450**	.689**	.536**	.609**	.756**
C3-NAP	.807**	.635**	.513**	.840**	.844**	.699**	.677**	.757**	.739**	.733**	.654**	.438**	.679**	.519**	.585**	.741**
C4-NAP	.678**	.753**	.550**	.791**	.829**	.824**	.757**	.812**	.790**	.783**	.757**	.524**	.749**	.595**	.603**	.788**
C1-PHE	.775**	.736**	.544**	.844**	.890**	.813**	.781**	.840**	.813**	.812**	.775**	.537**	.762**	.619**	.634**	.821**
C2-PHE	.756**	.728**	.558**	.825**	.868**	.794**	.772**	.833**	.802**	.801**	.750**	.526**	.756**	.600**	.619**	.805**
C3-PHE	.785**	.716**	.542**	.835**	.873**	.784**	.766**	.824**	.805**	.803**	.748**	.540**	.754**	.600**	.612**	.803**
C4-PHE	.240**	.213*	.328**	.214*	.203*	0.121	.239**	.235**	.186*	.239**	0.172	.221*	.196*	0.133	0.045	0.13
2-M-ANT	.309**	.366**	.312**	.344**	.381**	.409**	.401**	.408**	.363**	.357**	.367**	.218*	.347**	.216*	.327**	.373**
C1-FLU	.755**	.739**	.559**	.827**	.857**	.801**	.750**	.816**	.798**	.788**	.742**	.514**	.755**	.593**	.653**	.804**
C1-FLA	.753**	.711**	.593**	.804**	.848**	.762**	.787**	.832**	.786**	.792**	.742**	.549**	.746**	.591**	.612**	.786**
C1-PYR	.727**	.743**	.602**	.791**	.833**	.778**	.784**	.831**	.794**	.796**	.743**	.564**	.759**	.595**	.600**	.770**
C1-CHR	.758**	.792**	.609**	.819**	.852**	.833**	.775**	.838**	.813**	.794**	.774**	.566**	.809**	.646**	.754**	.859**
7-M-BaP	.632**	.624**	.497**	.655**	.666**	.661**	.623**	.687**	.658**	.594**	.537**	.383**	.626**	.493**	.686**	.663**

Continued

Compounds s	C1-NAP	C2-NAP	C3-NAP	C4-NAP	C1-PHE	C2-PHE	C3-PHE	C4-PHE	2M-ANT	C1-FLU	C1-FLA	C1-PYR	C1-CHR	7M-BaP
C1-NAP	1													
C2-NAP	.990**	1												
C3-NAP	.972**	.991**	1											
C4-NAP	.862**	.914**	.924**	1										
C1-PHE	.931**	.966**	.966**	.961**	1									
C2-PHE	.920**	.960**	.968**	.966**	.993**	1								
C3-PHE	.928**	.955**	.954**	.939**	.974**	.972**	1							
C4-PHE	.309**	.301**	.316**	.282**	.287**	.309**	.293**	1						
2M-ANT	.557**	.573**	.566**	.606**	.611**	.617**	.597**	.410**	1					
C1-FLU	.927**	.961**	.965**	.975**	.980**	.984**	.957**	.303**	.635**	1				
C1-FLA	.911**	.942**	.942**	.940**	.972**	.978**	.963**	.330**	.669**	.964**	1			
C1-PYR	.892**	.932**	.941**	.963**	.965**	.977**	.950**	.362**	.659**	.975**	.975**	1		
C1-CHR	.876**	.904**	.901**	.925**	.929**	.935**	.909**	.237**	.586**	.956**	.920**	.918**	1	
7M-BaP	.781**	.804**	.814**	.811**	.788**	.818**	.778**	.247**	.550**	.843**	.813**	.841**	.879**	1

*Correlation is significant at the 0.05 level (two-tailed)

**Correlation is significant at the 0.01 level (two-tailed)

Table S5 TEQ_{BaP} of 16 PAHs and APAHs in topsoil of different functional areas in Huaibei Coalfield.

Compounds	TEF	Mining area (n=15)		Industrial area (n=61)		Residential area (n=6)		Agricultural area (n=45)	
		16PAHs	APAHs	16PAHs	APAHs	16PAHs	APAHs	16PAHs	APAHs
NAP/ANAPs	0.001	0.17	2.56	0.08	1.01	0.05	0.32	0.03	0.20
ACY	0.001	0.01	—	0.01	—	0.01	—	0.01	—
ACE	0.001	0.02	—	0.02	—	0.01	—	0.01	—
FLU/C1-FLU	0.001	0.09	0.09	0.04	0.05	0.02	0.02	0.02	0.01
PHE/APHEs	0.001	0.30	2.07	0.17	1.23	0.06	0.43	0.04	0.30
ANT/2M-ANT	0.01	0.15	0.03	0.14	0.04	0.06	0.01	0.06	0.01
FLA/C1-FLA	0.001	0.06	0.15	0.11	0.10	0.03	0.02	0.02	0.01
PYR/C1-PYR	0.001	0.08	0.21	0.09	0.16	0.03	0.04	0.01	0.03
BaA	0.1	3.71	—	3.27	—	1.10	—	1.09	—
CHR/C1-CHR	0.01	1.04	1.39	0.97	0.63	0.31	0.14	0.15	0.10
BbF	0.1	5.46	—	7.29	—	1.03	—	1.04	—
BkF	0.1	1.79	—	3.05	—	1.79	—	1.49	—
BaP/7M-BaP	1	41.87	67.73	47.01	47.49	12.94	9.87	11.30	16.04
InP	0.1	1.63	—	1.88	—	1.30	—	1.30	—
DBA	1	82.49	—	66.02	—	57.50	—	57.50	—
BgP	0.01	0.60	—	0.46	—	0.15	—	0.13	—
ΣTEQ_{BaP}	—	139.46	74.23	130.61	50.71	76.39	10.87	74.18	16.70

Cancer risks (CRs)

The cancer risks (CRs) were estimated using the following Eqs. (1) and (2) and (3), respectively (USEPA, 2004).

$$CR_{ing} = \frac{\mathrm{Csoil} \times \mathrm{IRsoil} \times \mathrm{EF} \times \mathrm{ED} \times \mathrm{CF}}{\mathrm{BW} \times \mathrm{AT}} \times CSF_{ing} \quad (1)$$

where CR_{ing} is the cancer risk (unitless) through ingestion of soil particles. C_{soil} is the total BaP_{eq} concentrations of soil PAHs ($mg{\cdot}kg^{-1}$); IR_{soil} is the soil ingestion rate (mg d^{-1}); EF is the exposure frequency (d $year^{-1}$), ED is the exposure duration (years); CF is conversion factor of 10^{-6} kg mg^{-1}; BW is body weight (kg), AT is the average life span (d), CSF_{ing} is ingestion cancer slope factor ((mg kg^{-1} $d^{-1})^{-1}$).

$$CR_{derm} = \frac{\mathrm{Csoil} \times \mathrm{SA} \times \mathrm{AFsoil} \times \mathrm{ABS} \times \mathrm{EF} \times \mathrm{ED} \times \mathrm{CF}}{\mathrm{BW} \times \mathrm{AT}} \times \frac{\mathrm{CSFderm}}{\mathrm{GIABS}} \quad (2)$$

where CR_{derm} is the cancer risk (unitless) for dermal contact pathway; SA is the exposed surface area of the skin (cm^2), AF_{soil} is the dermal adherence factor (mg cm^2), ABS is absorption factor (unitless), CSF_{derm} is ingestion cancer slope factor ((mg kg^{-1} $d^{-1})^{-1}$); and GIABS is fraction of contaminant absorbed in gastrointestinal tract (unitless).

$$CR_{inh} = \frac{\mathrm{Csoil} \times \mathrm{IRair} \times \mathrm{EF} \times \mathrm{ED}}{\mathrm{PEF} \times \mathrm{BW} \times \mathrm{AT}} \times CSF_{inh} \quad (3)$$

where CR_{inh} is the cancer risk (unitless) for inhalation pathway. IR_{air} is the inhalation rate (m^3 d^{-1}), CSF_{inh} is inhalation cancer slope factor ((mg kg^{-1} $d^{-1})^{-1}$), PEF is the soil particle emission factor (m^3 kg^{-1}).

$$CR_{total} = CR_{ing} + CR_{derm} + CR_{inh} \quad (4)$$

The total cancer risk (CR_{total}) was calculated by summing the CR of three exposure ways. And the parameters of these variables used in the above-mentioned equations are given in Table S5.

Table S6 Parameters used in the incremental lifetime cancer risk assessment [31].

Exposure variable	Unit	Child	Adult	Reference
Body weight (BW)	kg	16.2	61.8	US EPA (2011)
Exposure frequency (EF）	day $year^{-1}$	180	180	Ferreira-Baptista and De Miguel (2005)
Exposure duration (ED）	year	6	30	US EPA (2011)
Inhalation rate (IR_{air})	m^3 day^{-1}	7.6	20	US EPA (2011)
Soil ingestion rate (IR_{soil})	mg day^{-1}	200	100	US EPA (2011)
Dermal expore area (SA)	cm^2 day^{-1}	2800	5700	US EPA (2011)
Dermal adherence factor (AF)	mg cm^{-2}	0.7	0.07	US EPA (2011)
Dermal adsorption fraction (ABS)	Unitless	0.1	0.1	US EPA (2001)
Gastrointestinal absorption factor (GIABS)	Unitless	1	1	US EPA (2011)
Average life span (AT)	day	70 × 365 = 25550	70 × 365 = 25550	Ferreira-Baptista and De Miguel (2005)
Ingestion cancer slope factor (CSF_{ing})	(mg kg^{-1} $day^{-1})^{-1}$	7.3	7.3	Knafla et al., 2006
Inhalation cancer slope factor (CSF_{inh})	(mg kg^{-1} $day^{-1})^{-1}$	3.85	3.85	Wang, 2007
Dermal cancer slope factor (CSF_{der})	(mg kg^{-1} $day^{-1})^{-1}$	25	25	US EPA, 1994
Particle emission factor (PEF)	m^3 kg^{-1}	1.36×10^9	1.36×10^9	US EPA (2011)

Reference

60. Liu, J.; Liu, G.; Zhang, J.; Yina, H.; Wang, R., Occurrence and risk assessment of polycyclic aromatic hydrocarbons in soil from the Tiefa coal mine district, Liaoning, *China. J. Environ. Monit.* **2012,** 14, 2634–2642.
61. Nam, J. J.; Song, B. H.; Eom, K. C.; Lee, S. H.; Smith, A., Distribution of polycyclic aromatic hydrocarbons in agricultural soils in South Korea. *Chemosphere.* **2003,** 50, 1281–1289.
62. Yin, C. Q.; Jiang, X.; Yang, X. L.; Bian, Y. R.; Wang, F., Polycyclic aromatic hydrocarbons in soils in the vicinity of Nanjing, China. *Chemosphere.* **2008,** 73 (3), 389-94.
63. Agarwal, T.; Khillare, P. S.; Shridhar, V.; Ray, S., Pattern, sources and toxic potential of PAHs in the agricultural soils of Delhi, India. *J. Hazard. Mater.* **2009,** 163 (2-3), 1033-9.
64. Hao, R.; Wan, H.-F.; Song, Y.-T.; Jiang, H.; Peng, S.-L., Polycyclic Aromatic Hydrocarbons in Agricultural Soils of the Southern Subtropics, China. *Pedosphere.* **2007,** 17 (5), 673-680.
65. Maliszewska-Kordybach, B.; Smreczak, B.; Klimkowicz-Pawlas, A., Effects of anthropopressure and soil properties on the accumulation of polycyclic aromatic hydrocarbons in the upper layer of soils in selected regions of Poland. *Appl. Geochem.* **2009,** 24 (10), 1918-1926.
66. Yang, J.; Sun, P.; Zhang, X.; Wei, X. Y.; Huang, Y. P.; Du, W. N.; Qadeer, A.; Liu, M.; Huang, Y., Source apportionment of PAHs in roadside agricultural soils of a megacity using positive matrix factorization receptor model and compound-specific carbon isotope analysis. *J. Hazard. Mater.* **2021,** 403, 123592.
67. Liao, X.; Wu, Z.; Li, Y.; Cao, H.; Su, C., Effect of various chemical oxidation reagents on soil indigenous microbial diversity in remediation of soil contaminated by PAHs. *Chemosphere.* **2019,** 226, 483-491.

68. Jiao, W.; Lu, Y.; Li, J.; Han, J.; Wang, T.; Luo, W.; Shi, Y.; Wang, G., Identification of sources of elevated concentrations of polycyclic aromatic hydrocarbons in an industrial area in Tianjin, China. *Environ. Monit. Assess.* **2009,** 158 (1-4), 581-592.
69. Idowu, O.; Semple, K. T.; Ramadass, K.; O'Connor, W.; Hansbro, P.; Thavamani, P., Analysis of polycyclic aromatic hydrocarbons (PAHs) and their polar derivatives in soils of an industrial heritage city of Australia. *Sci. Total Environ.* **2020,** 699, 1-12.
70. Jia, T.; Guo, W.; Xing, Y.; Lei, R.; Wu, X.; Sun, S.; He, Y.; Liu, W., Spatial distributions and sources of PAHs in soil in chemical industry parks in the Yangtze River Delta, China. *Environ. Pollut.* **2021,** 283, 117121.
71. Jiang, Y.; Yves, U. J.; Sun, H.; Hu, X.; Zhan, H.; Wu, Y., Distribution, compositional pattern and sources of polycyclic aromatic hydrocarbons in urban soils of an industrial city, Lanzhou, China. *Ecotoxicol. Environ. Saf.* **2016,** 126, 154-162.
72. Li, Y.; Long, L.; Ge, J.; Yang, L. X.; Cheng, J. J.; Sun, L. X.; Lu, C.; Yu, X. Y., Presence, distribution and risk assessment of polycyclic aromatic hydrocarbons in rice-wheat continuous cropping soils close to five industrial parks of Suzhou, China. *Chemosphere.* **2017,** 184, 753-761.
73. Sushkova, S.; Minkina, T.; Deryabkina, I.; Rajput, V.; Antonenko, E.; Nazarenko, O.; Yadav, B. K.; Hakki, E.; Mohan, D., Environmental pollution of soil with PAHs in energy producing plants zone. *Sci. Total Environ.* **2019,** 655, 232-241.
74. Liu, Y.; Gao, P.; Su, J.; da Silva, E. B.; de Oliveira, L. M.; Townsend, T.; Xiang, P.; Ma, L. Q., PAHs in urban soils of two Florida cities: Background concentrations, distribution, and sources. *Chemosphere.* **2019,** 214, 220-227.
75. Thiombane, M.; Albanese, S.; Di Bonito, M.; Lima, A.; Zuzolo, D.; Rolandi, R.; Qi, S.; De Vivo, B., Source patterns and contamination level of polycyclic aromatic hydrocarbons (PAHs) in urban and rural areas of Southern Italian soils. *Environ. Geochem. Health.* **2019,** 41 (2), 507-528.
76. Qu, C.; Albanese, S.; Lima, A.; Hope, D.; Pond, P.; Fortelli, A.; Romano, N.; Cerino, P.; Pizzolante, A.; De Vivo, B., The occurrence of OCPs, PCBs, and PAHs in the soil, air, and bulk deposition of the Naples metropolitan area, southern Italy: Implications for sources and environmental processes. *Environ. Int.* **2019,** 124, 89-97.
77. Gao, P.; Xu, M.; Liu, Y.; da Silva, E. B.; Xiang, P.; Ma, L. Q., Emerging and legacy PAHs in urban soils of four small cities: Concentrations, distribution, and sources. *Sci. Total Environ.* **2019,** 685, 463-470.
78. Tang, L.; Tang, X. Y.; Zhu, Y. G.; Zheng, M. H.; Miao, Q. L., Contamination of polycyclic aromatic hydrocarbons (PAHs) in urban soils in Beijing, China. *Environ. Int.* **2005,** 31 (6), 822-828.
79. Jiang, Y.; Wang, X.; Wang, F.; Jia, Y.; Wu, M.; Sheng, G.; Fu, J. Levels, composition profiles and sources of polycyclic aromatic hydrocarbons in urban soil of Shanghai, China. *Chemosphere.* **2009,** 75 (8), 1112-1118.
80. Bortey-Sam, N.; Ikenaka, Y.; Nakayama, S. M. M.; Akoto, O.; Yohannes, Y. B.; Baidoo, E.; Mizukawa, H.; Ishizuka, M., Occurrence, distribution, sources and toxic potential of polycyclic aromatic hydrocarbons (PAHs) in surface soils from the Kumasi Metropolis, Ghana. *Sci. Total Environ.* **2014,** 496, 471-478.
81. Parra, Y. J.; Oloyede, O. O.; Pereira, G. M.; de Almeida Lima, P. H. A.; da Silva Caumo, S. E.; Morenikeji, O. A.; de Castro Vasconcellos, P., Polycyclic aromatic hydrocarbons in soils and sediments in Southwest Nigeria. *Environ. Pollut.* **2020,** 259, 113732.
82. Zuo, Q.; Duan, Y.; Yang, Y.; Wang, X. J.; Tao, S., Source apportionment of polycyclic aromatic hydrocarbons in surface soil in Tianjin, China. *Environ. Pollut.* **2007,** 147 (2), 303-310.
83. Wang, Z.; Chen, J.; Qiao, X.; Yang, P.; Tian, F.; Huang, L. Distribution and sources of polycyclic aromatic hydrocarbons from urban to rural soils: a case study in Dalian, China. *Chemosphere.* **2007,** 68 (5), 965-971.
84. Liang, M.; Liang, H.; Rao, Z.; Hong, X., Characterization of polycyclic aromatic hydrocarbons in urban-rural integration area soil, North China: Spatial distribution, sources and potential human health risk assessment. *Chemosphere.* **2019,** 234, 875-884.
